# 

**Published:** 1888-09-29

**Authors:** 


### To Residents, Secretaries, and Matrons.

The Editor will be glad to have the Regulations and each Report as issued by Asylums, Hospitals, Convalescent and Nursing Institutions, as well as those of other Charities, and an early copy in duplicate of all Appeals and Festival Notices, etc., addressed to *The Editor, The Lodge, Porchester Square, London, W.* Any superintendent, secretary, matron, or other chief official who regularly sends such information may be registered, on application to the Publisher, to receive free of cost a cover for binding The Hospital in half-yearly volumes.

### Notice to Advertisers and Subscribers.

All Advertisements and Business Communications should be addressed to The Publisher, *not to the Editor*, The Hospital, 38, Old Jewry, E.C.
A Scale of Charges will be found in the Advertisement Sheets, page iii.

Alcohol as a Remedial Agent.—At the meeting of the German physicians known as the Wiesbaden Congress, Dr. Binz, of Bonn, read a paper on "Alcohol as a Remedial Agent," in which he referred to expressed opinions and upheld the efficacious virtues of alcohol, contending that it has an especial value in heart-failure and lung-disease, that it is consumed in the organism, and that it operates as a controller of pyrexia and fever. While he regards it as an invaluable aid to the physician, Dr. Binz at the same time holds that the consumption of alcohol between meals, especially in the form of beer, is in Germany a "national evil."

### Amusements with Profit for all Ages.

**SPECIAL NOTICE.—A New Prize Scheme will be presented next month, in which we hope all past, and many new, competitors will take part.**

### THE CHILDREN'S CORNER.

**PRIZES.**

**FOR MOST CORRECT ANSWERS TO PUZZLES.**

This Commenced October 1st, 1887.

One Pound a Month.

**A PRIZE OF THREE GUINEAS**

to the Competitor who shall have sent in the largest number of correct or best answers during the twelve months.

### Fifth Week Puzzles.

No 17. Double Acrostic.

1. The reverse of old. 2. Space. 3. A deep hole. 4. A number. 5. To linger. 6. A fish. 7. A river of South America. 8. A native of Africa. Initials. give the name of a famous military leader, finals the battle which caused his overthrow.

No. 18. Enigma.

I have no head, and a tail I lack,
But oft have arms and legs and back ;
I inhabit the tavern, the palace, the cot,
'Tis a beggarly residence where I am not.
If a monarch were present (I tell you no fable),
I still should be placed at the head of the table.

No 19. Flowers Enigmatically Expressed.

1. A biped, whose garments are tattered and torn ;
2. And the flower which treats country cousins with scorn,
3. The flower which supplies us with heat and with light,
4. And the one whose part differs as sunshine and night.

No. 20. Geographical Charade.

My *first* is an island : my *second* the name of an old English city, my *whole* is a town noted for its cotton manufactories.

### Results of Third Week Puzzles.

No. 9. Double Acrostic.

M ont Blan C

A jacci O

L abrado R

T af F

A njo U

No. 10. Square Word.

C R A G
R A T E
A T O M
G E M S

No. 11. Enigma.—The gentleman had seven children, six sons and one daughter.

No. 12. Geographical Charade.—Peterhead = Peterhead.

| Marks. | Sept. 15th. | Total First, Second, and Third Weeks. |
|---|---|---|
| A. C. K. | 4 | 11 |
| Gem | 4 | 10 |
| G. M. M. | 4 | 8 |
| Scrooge | 4 | 7 |
| Thanet | 4 | 11 |
| Tynesider | — | 3 |
| Judy | 3 | 6 |
| Primrose | 4 | 11 |
| J. W. M. | 4 | 8 |
| Agamemnon | 4 | 12 |
| G. E. R. | 4 | 10 |
| Happy Eliza | 3 | 6 |
| Penelope | 4 | 8 |
| Boadicea | 4 | 10 |
| Ikye Mo | 1 | 2 |
| Poodle | 2 | 2 |

N.B.—*The Children's Corner will be discontinued after this week.*

### Conditions.

I. Every paper sent in must be clearly written, and one side only must be used. All competitors must mark at the head of their papers the number of their competition.

II. Each paper must be signed by the author with his or her real name and address. A *nom de plume* may be added if the writer does not desire to be referred to by us by his real name. In the case of all prizewinners, however, the real name and address will be published.

III. All communications relating to prize competitions should be addressed to "The Prize Editor, The Hospital, 38, Old Jewry, London, E.C." Competitors are requested not to include in letters, etc., to the Prize Editor, communications on matters of other business. All letters must be fully prepaid.

IV. The Prize Editor of The Hospital is the sole judge of the competitions, and his decision is in all cases final and without appeal.

V. The Prize Editor reserves the right to publish any paper sent in, whether it receives a prize or not. He does not hold himself responsible for any MSS. sent, nor can he undertake to return rejected competitions.

VI.—All children resident at school are requested to forward their *home* address in addition to the school one, when entering for the "Children's Corner" Competitions.

